# An Efficient Bayesian Approach to Exploit the Context of Object-Action Interaction for Object Recognition

**DOI:** 10.3390/s16070981

**Published:** 2016-06-25

**Authors:** Sungbaek Yoon, Hyunjin Park, Juneho Yi

**Affiliations:** 1 School of Electronic and Electrical Engineering, Sungkyunkwan University, Suwon 16419, Korea; beagii@skku.edu (S.Y.); hyunjinp@skku.edu (H.P.); 2 School of Information and Communication Engineering, North University of China, Taiyuan 03000, China; jhyi@skku.edu

**Keywords:** object recognition, object-action context, object-human interaction

## Abstract

This research features object recognition that exploits the context of object-action interaction to enhance the recognition performance. Since objects have specific usages, and human actions corresponding to these usages can be associated with these objects, human actions can provide effective information for object recognition. When objects from different categories have similar appearances, the human action associated with each object can be very effective in resolving ambiguities related to recognizing these objects. We propose an efficient method that integrates human interaction with objects into a form of object recognition. We represent human actions by concatenating poselet vectors computed from key frames and learn the probabilities of objects and actions using random forest and multi-class AdaBoost algorithms. Our experimental results show that poselet representation of human actions is quite effective in integrating human action information into object recognition.

## 1. Introduction

Object recognition is difficult due to a variety of factors, including viewpoint variation, illumination changes, occlusion, etc. However, before encountering these factors, the inherent difficulty of object recognition lies in the fact that there is a large amount of intra-category appearance variation, and objects from different categories may have similar appearances. In order to improve the performance of object recognition, researchers have exploited contextual information that includes spatial [1,2,3], semantic [4,5,6,7], and scale [8,9] contexts. Spatial context refers to information about the potential locations of objects in images or the positional relationship between objects. Semantic context provides clues related to the co-occurrence of objects with other objects in a scene. Scale context gives the relative scale of objects in a scene.

**Figure 1:**
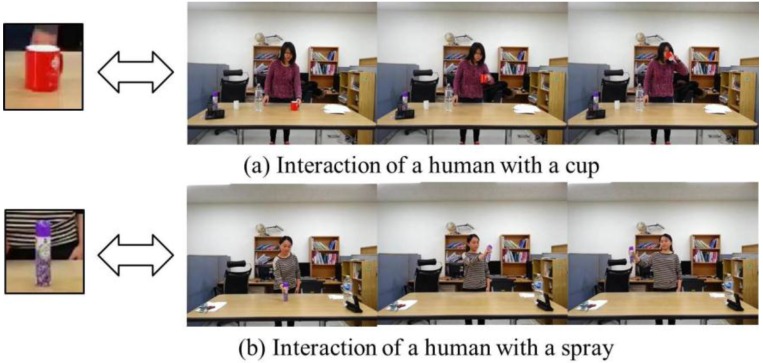
Examples of object-action context. Objects have specific usages and human actions corresponding to these usages can be related to these objects.

In this work, we focus on the context of object-action interaction, which has been relatively unexplored. Since objects have specific usages, and human actions corresponding to these usages can be related to these objects, it is possible to improve the performance of object recognition by exploiting human interactions with objects as a type of context information. Especially, when objects from different categories have similar appearances, analyzing the human action associated with each object can be effective in resolving the ambiguity related to recognizing objects. As illustrated in Figure 1, when a cup or spray bottle is held by a human hand, they look very similar because of their cylindrical structures. In this case, exploiting the context of the object-action interaction greatly facilitates the distinction between the two objects.

There have been a few experiments that have adopted similar ideas with different representations of human actions, objects, and computational algorithms. Moore et al. [10] depicted human actions using the hidden Markov model (HMM) by tracking hand locations, although it is not easy to normalize different action speeds for different individuals. Gupta et al. [11] recognized human-object interactions based on the integration of action recognition and object recognition, where human actions and objects contribute mutual contexts for each other.

They also represented human actions using HMM by detecting hand trajectories. Human actions were segmented into several atomic actions; however, stable segmentation of each action into atomic actions is difficult. Yao et al. [12] modeled the context between human poses and objects using Markov random field modeling to recognize human-object interactions. Their work is based on a single pose, and it is not clear which pose belongs to which action. Thus, categories of objects may be relatively obscured compared to when action information is employed. Grabner et al. [13] described the relations between objects and a human pose based on matching the shapes of them. They exploited the relations to detect an affordance which is functionality implied by objects rather than recognizing a specific object category.

Alternatively, deep learning approaches, such as convolutional neural networks (CNN) [14], have achieved great success in object recognition. However, as can be seen in the experimental results, when there are not enough labelled images available, the recognition performance is not as high as expected. In addition, it is difficult to find an optimal CNN architecture for a given problem.

The goal of this study is to efficiently and effectively incorporate human action information into object recognition in order to boost the recognition performance. We employ a few image frames that contain key poses, which can be used to distinguish human actions. Since an assemblage of key poses can take advantage of the fiducial appearance of the human body in action, representation of human actions by concatenating a few key poses is quite effective. The main contribution of this work is the establishment of an effective Bayesian approach that exploits the probabilities of objects and actions, through random forest and multi-class AdaBoost algorithms.

**Figure 2:**
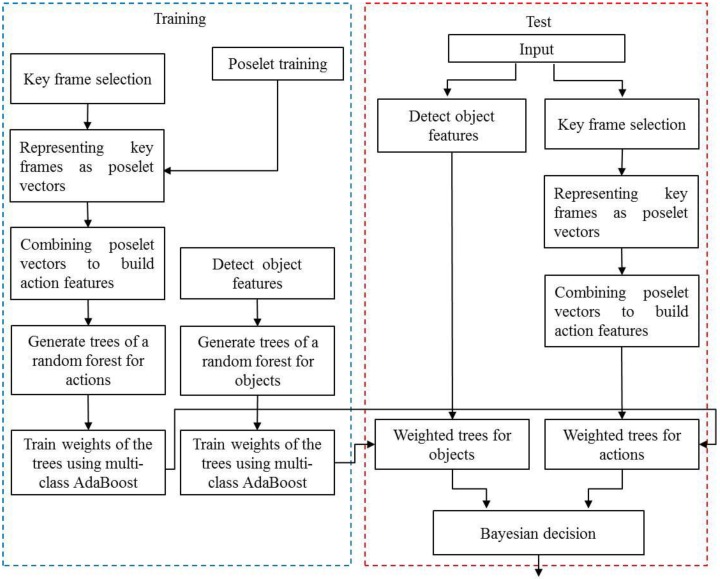
Object recognition using object-action context.

Figure 2 overviews our method, which recognizes objects using object-action context. First, random forests for objects and actions are trained independently using object features obtained from object images and action features acquired from video sequences. Additionally, by regarding each tree in a random forest as a weak classifier, the weight of the tree is determined using multi-class AdaBoost [15]. The object categories of the input data are determined by applying a Bayesian approach using the probabilities calculated from object features and action features. We represent human actions by concatenating poselet vectors [16,17] computed from key frames in a video. poselets depicting local parts of human poses are feature vectors that are strictly clustered based on their appearance. The value of an element in a poselet vector is the maximum response value of the key frame to each poselet; we use a support vector machine (SVM) as the poselet classifier. Recently, with the resurgence of the neural network, poselets have a new version based on the neural network [18]. However, the neural network-based approach is more computationally expensive than our random forest-based method and also requires many more training images to produce a well-trained network. We use the histogram of oriented gradients (HOG) to represent objects. The experimental results show that our method, using object-action context, enhances the performance of object recognition when the appearances of objects belonging to the same category largely differ and objects of different categories are similar in appearance.

This paper is organized as follows. The following section presents the probabilistic model we propose for object recognition and describes our approach for determining the probabilities of objects and human actions using random forest and multi-class AdaBoost algorithms. The methods used for representing objects and actions are given in Section 3, and our experimental results are reported in Section 4.

## 2. Incorporating Object-Action Context into Object Recognition

O and A denote object categories and human action categories, respectively. $x^{O}$ is an appearance feature from an object, and $x^{A}$ is a feature of a human action related to the object. Given $x^{O}$and $x^{A}$, the probability of the object category, $p\left(O|x^{O},x^{A}\right)$, can be depicted by Equation (1):

(1) $$\begin{array}{ll} p\left(O|x^{O},x^{A}\right) & =p\left(O|x^{O}\right)p\left(O|x^{A}\right)\\ & =p\left(O|x^{O}\right)\sum_{A}p\left(O,A|x^{A}\right)\\ & =\;p\left(O|x^{O}\right)\sum_{A}p\left(O|A\right)p\left(A|x^{A}\right) \end{array}$$

Our method outputs the object that maximizes $p\left(O|x^{O},x^{A}\right)$ as the recognition result. The goal of this method is to efficiently learn the probability of the object category $p\left(O|x^{O}\right)$ given an object feature $x^{O}$, the probability of the action category $p\left(A|x^{A}\right)$ given an action feature $x^{A}$, and p(O|A).

**Figure 3:**
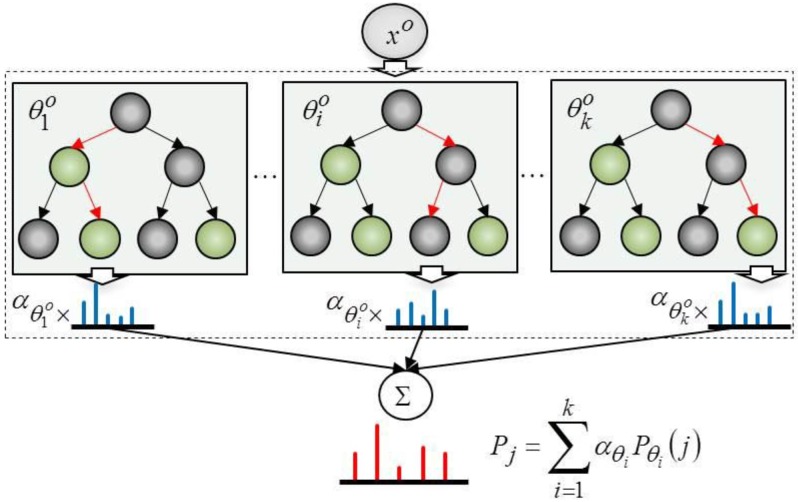
Computing the probabilities of object categories using a random forest and multi-class Adaboost.

We first describe how to estimate $p\left(O|x^{O}\right)$ and $p\left(A|x^{A}\right)$. We employ a random forest to learn $p\left(O|x^{O}\right)$ and $p\left(A|x^{A}\right)$. Figure 3 depicts the process used to calculate the probability of the object categories. The probability of object category, $P_{j}$, is a weighted summation of the probabilities of the object categories, $P_{\theta_{i=1,\ldots ,n}}\left(j\right)$, which are obtained from trees in the random forest. The weights of the trees, $\alpha_{\theta_{i=1,\ldots ,n}^{O}}$, are trained by multi-class Adaboost.

The training process for the probability of an object category is as follows. First, given training data, $D=\left\{\left(x^{o_{1}},o_{1}\right),\ldots ,\left(x^{o_{n}},o_{n}\right)\right\}$ and $x^{o_{j}}=\left\{x_{1}^{o_{j}},\ldots ,x_{M_{i}}^{o_{j}}\right\}$, a random forest of k trees, $\theta_{1},\ldots ,\theta_{k}$, is generated from the data. $x_{i}^{o_{j}}$ represents the $i^{\text{th}}$ object feature belonging to object category, $o_{j}$. The probability of $o_{j}$ that is computed by the random forest is given in Equation (2):

(2) $$p\left(o_{j}|x^{O}\right)\equiv p\left(o_{j}|x^{O},\Theta^{O}\right)=\frac{1}{\left|\Theta^{O}\right|}\sum_{i=1}^{k}\alpha_{\theta_{i}^{O}}\frac{n_{o_{j},\theta_{i}^{O}}}{n_{\theta_{i}^{O}}}$$

Here, $\Theta^{O}$ is the random forest built from the object features where $\theta_{i}^{O}\in \Theta$ denotes the $i^{\text{th}}$ decision tree and $\left|\Theta^{O}\right|=k$. $n_{o_{j},\theta_{i}^{O}}$ represents the amount of training data, which is classified as the object category, $o_{j}$. Finally, $n_{\theta_{i}^{O}}$ is the total amount of training data in tree $\theta_{i}^{O}$ at the leaf node. By treating each tree in the random forest as a weak classifier, the weight of each tree, $\alpha_{\theta_{i}^{O}}$, is learned using multi-class Adaboost [15]. $p\left(A|x^{A}\right)$ is determined in the exact same way as above by using action features.

For splitting nodes in the trees of the random forests, two parameters, ‘MinParentSize’ and ‘MinLeafSize’, are defined. ‘MinParentSize’ and ‘MinLeafSize’ denote the number of samples in a node and the number of samples in a leaf node, respectively. We have set ‘MinParentSize’ to 20 and ‘MinLeafSize’ to 10. A tree stops splitting when any of the following conditions hold: (1) if a node contains only samples of one class; (2) the number of samples is fewer than ‘MinParentSize’ samples in a node; and (3) any split applied to a node generates children with smaller than ‘MinLeafSize’ samples.

**Figure 4:**
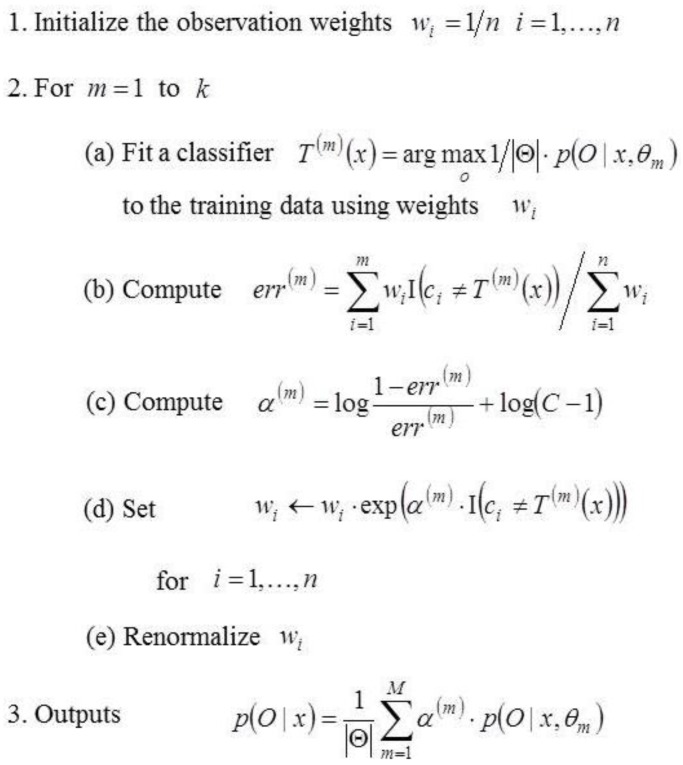
Training the weight of each tree in the random forest using multi-class Adaboost.

Figure 4 describes the learning process of multi-class Adaboost, which is an extension of the binary Adaboost learning process into multi-classes. It generates classification rules and readjusts the distribution of the training data using the preceding classification rules. When the amount of training data is n and C is the number of categories, the initial distribution of the data is computed in the first step. During k repetitions, w is updated and data that are not well-classified are assigned higher values. In the second step, the error of the weak classifier, $T^{\left(m\right)}\left(x\right)$, is computed and w is renewed based on the error. Lastly, we acquire the probability of an object category as a linear combination of the probabilities obtained from the trees that are weak classifiers; this is done using the weight α. In Step 2c, the extra term, log(C−1), represents the only variation from the binary Adaboost algorithm. Unlike in binary classification, where the error rate of random guessing is 1/2, the error rate of random guessing is (C−1)/C for multi-classification. The Adaboost assumption, which expects that the error rate of the weak classifier is less than 1/2, is not satisfied. Thus, in order to solve this drawback of Adaboost, the log(C−1) term is added.

To estimate p(O|A), we use p(A|O) using the Bayesian rule:

(3) $$p\left(O|A\right)=\frac{p\left(A|O\right)p\left(O\right)}{\sum_{O}p\left(A|O\right)p\left(O\right)}$$

where p(A|O) can be calculated based on the number of observations associated with the same object category:

(4) $$p\left(A=a_{j}|O=o_{i}\right)=\frac{n_{j}}{N_{i}}$$

Here, $N_{i}$ is the number of observations associated with object category, $o_{i}$, and $n_{j}$ represents the number of observations for action category, $a_{j}$. In our experiments, training image sequences are collected such that each subject takes action that corresponds to the correct usage of a given object. Thus, in actual implementation, $p\left(A=a_{j}|O=o_{i}\right)=1$ for i=j; 0 otherwise. Here, i=j means an object and its correct action pair.

## 3. Representing Objects and Human Actions

We can regard human actions as an assemblage of continuous poses. However, on account of the similarity between the poses in adjacent frames, singling poses out from all of the video frames creates needless duplication. Thus, we extracted the key frames from the video in order to use the minimum number of poses to express human actions. We then deployed poselet vectors to represent the key frames.

**Figure 5:**
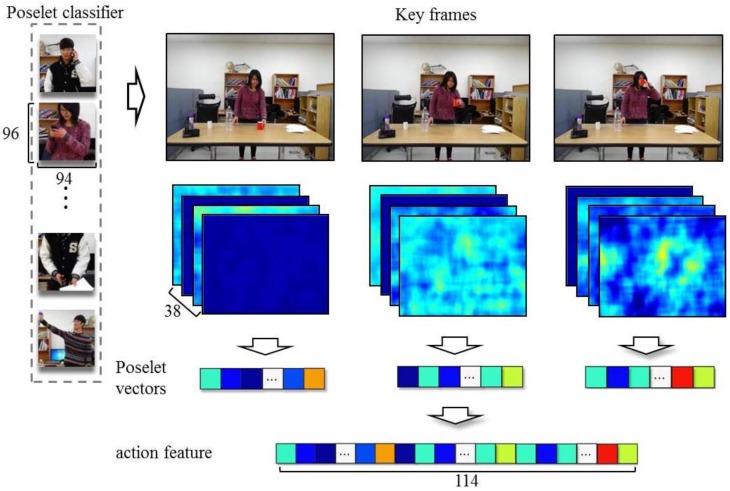
Creation of poselet vectors and action features.

**Figure 6:**
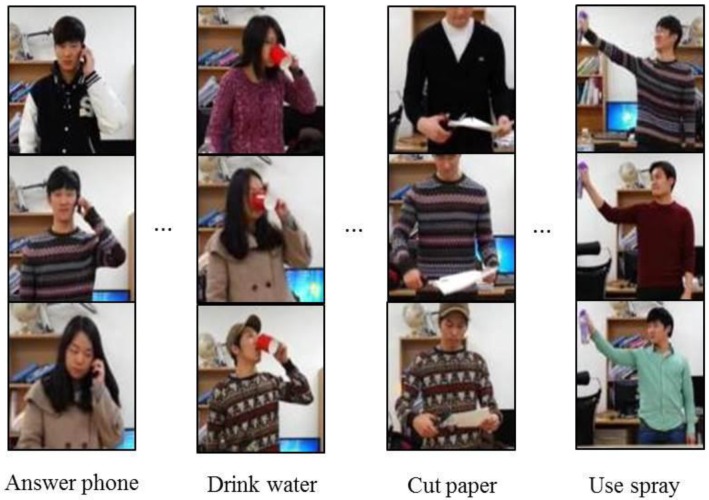
Examples of poselets.

Figure 5 shows the procedure used for turning key frames into poselet vectors. To describe the key frames using poselet vectors, the labeled poselets shown in Figure 6 are expressed by HOG [19], and an SVM is learned for each poselet. A poselet vector is generated using the maximum response values, which are obtained by applying all of the learned poselet SVMs to a key frame through a sliding window technique. An action feature is then obtained by concatenation of the poselet vectors.

To extract key frames from input video, a poselet vector is computed for each frame of the input video and the Euclidean distance between the frames (at a similar time as the training key frames) and the training key frames is computed using their poselet vectors. The frames with the minimum distance are selected as the key frames of the input video. Objects are represented using HOG. The size of an object image is 50×50.

## 4. Experimental Results

We compared the performance of our method with that of the one proposed by Gupta et al. [11] and a CNN. To our knowledge, the algorithm of Gupta et al. [11] is the most representative work that exploits human actions as context information for object recognition. We have included a CNN for performance comparison because CNN has recently achieved great success in object recognition.

We have also conducted an experiment using local space-time action features. To represent actions, we have used Bag of Visual words (BoV) model of local N-jets [20,21,22], which are built from space-time interest points (STIP) [21,22]. Local N-jets is one of the popular and strong motion features and its two first levels show velocity and acceleration. The code book for BoV is constructed using a K-means algorithm.

For our experiments, we designed a CNN architecture by referring to CIFAR10-demo [23]. As described in Table 1, the network contains 13 layers. The outputs of the first, second, and third convolutional layers are conveyed to the rectified linear unit (ReLU) and pooling layers. The first pooling layer is the max pooling layer and the remaining pooling layers are average pooling layers. The fourth convolutional layer and two fully-connected layers are linked to one another without interrupting the ReLU and pooling layers. The last fully-connected layer feeds its output to softmax.

For the experiments, we captured videos of 19 subjects performing four kinds of actions with four different objects (i.e., cups, scissors, phones, and spray bottles). Each of the subjects carried out actions using these objects. We constructed a dataset that contains 228 video sequences [24]. We extracted key frames from the video sequences in order to use the minimum number of poses to express human actions and deployed poselet vectors to represent the key frames. An action feature is represented as a concatenation of three poselet vectors. We used 38 kinds of poselets in this experiment. Thus, an action feature has 114 dimensions, to learn poselet SVMs, we used 20,308 positive images for 38 different poses and 2321 negative images. The size of a poselet training images is 96×94. A linear SVM is used to differentiate samples in a single poselet category from samples belonging to all of the remaining poselet categories.

In order to obtain more positive action data for the random forest and SVM, we used combinations of the frames adjacent to the key frames. As a result, to train the action random forest, we used the following amounts of action features: 1625 action features for the “drinking water” action, 7149 for “calling on the phone”, 1674 for “cutting paper”, and 678 for “spraying”. For training the multi-class AdaBoost, we used 848 action features for “drinking water”, 1890 for “calling on the phone”, 330 for “cutting paper”, and 658 for “spraying”.

**Figure 7:**
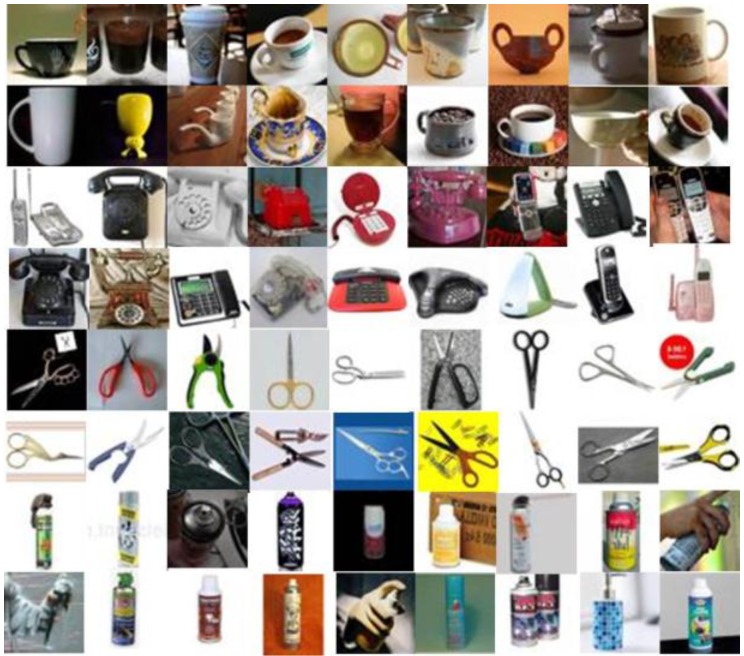
Some of the object images used in our experiments.

The object images used in the experiments were obtained from Google Image Search [25] and ImageNet [26]. We collected 3120 cup images, 4131 phone images, 2263 scissors images, and 2006 spray bottle images. We used 1200 images from each category to train the object random forest and 600 images for training the multi-class AdaBoost. Figure 7 shows some of the object images that were used in our experiments. The object image set contains objects that have a variety of appearances within the same category. Some objects, such as cups and sprays, are similar in appearance due to their cylindrical structure; however, these objects belong to different categories.

**Figure 8:**
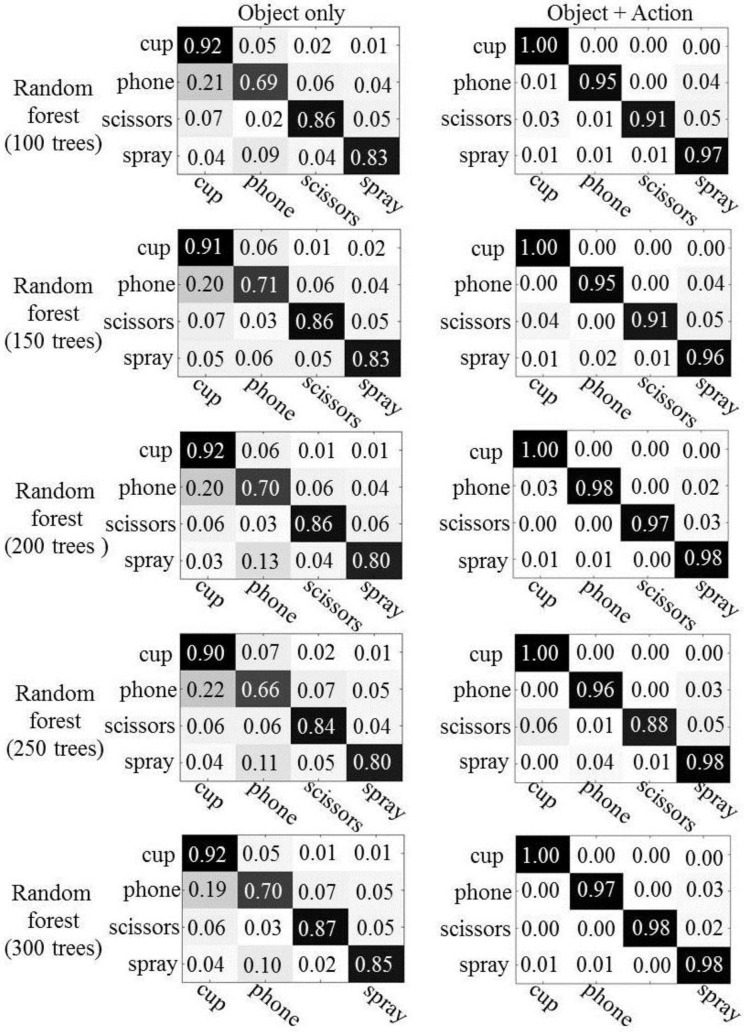
The results of object recognition: the left column shows the results using only the object’s appearance and the right column represents the results using the object’s appearance and human actions.

We conducted experiments with random forests using 100, 150, 200, 250, and 300 trees. Figure 8 shows the confusion matrices, which describe the results of object recognition. The first column represents the results of object recognition using only object appearance features and the second column depicts the results of object recognition using both object appearances and human actions. As expected, we see improved object recognition when using the human actions. Overall, the recognition rate is improved by between 4% (scissors) and 30% (phone), as compared to when only object appearances are used. The number of trees has little influence on the performance of object recognition in the experiments, both with and without human action context.

**Figure 9:**
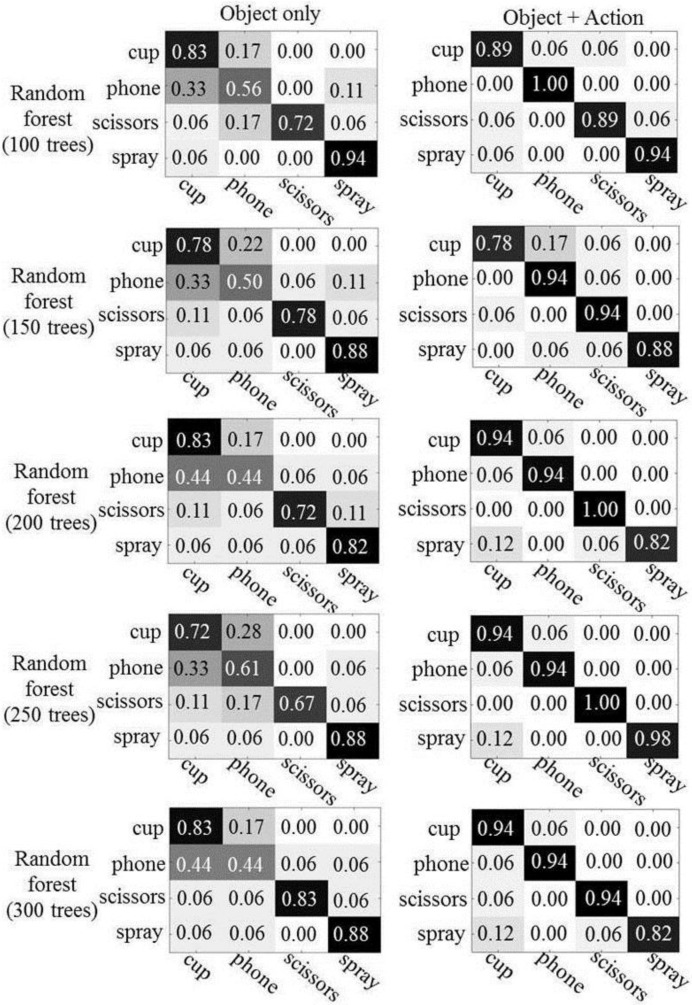
The results of object recognition using the BoV of Local N-jets: The first column shows the results using only the object’s appearance and the second column represents the results using the object’s appearance and human actions.

Figure 9 shows the result of object recognition in which actions are represented by the BoV of N-jets. For training the action random forest, we have used 39 action features for the “drinking water” action, 39 for “calling phone”, 39 for “cutting paper”, and 40 for “spraying”, respectively. For training the multi-class AdaBoost, the action features employed in the random forest are also utilized. For testing, we have used 18 action features for the “drinking water” action, 18 for “calling phone”, 18 for “cutting paper”, and 17 for “spraying”, respectively. We have used 1200 images from each object category to train the object random forest and 600 images for training the multi-class AdaBoost. For testing, we have used 18 object features for “cup”, 18 for “phone”, 18 for “scissors”, and 17 for “spray”, respectively.

Except for spray bottles, we have observed that the performance of object recognition is also significantly improved when using BoV of local N-jets as action features. The improvement of the recognition rate achieved ranges from 50% (phone) to 6% (cup). As described in Figure 8 and Figure 9, the poselet representations of the actions show better performances when recognizing cups, phones, and spray bottles. The differences of recognition rates between the action features were 6%–22% for cups, 1%–4% for phone, and 3%–16% for spray bottles. On the other hand, the recognition of the scissorss is improved from 3% to 12% using the BoV of local N-jets.

**Figure 10:**
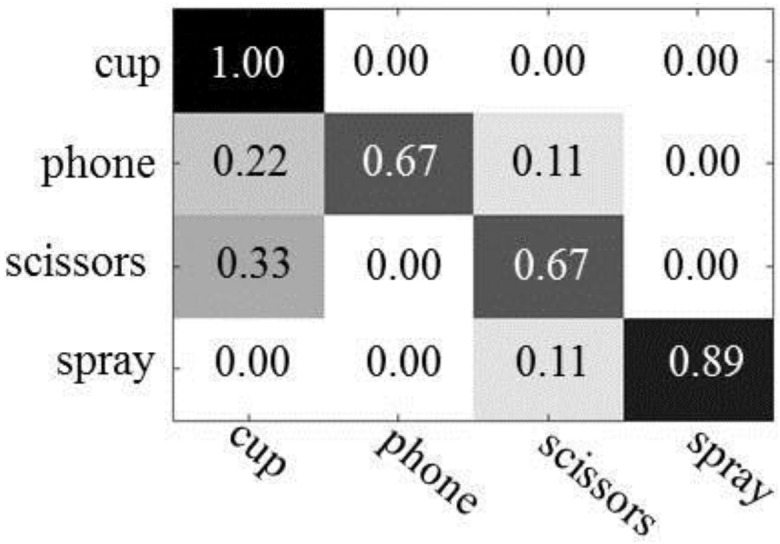
The results of Gupta’s algorithm for our experimental data.

Figure 10 shows the results of applying Gupta’s algorithm to our experimental data. With the exception of cups, objects exhibit low recognition performance compared with our method. The differences of recognition rates between our method and their method were 28%–31% for telephones, 21%–31% for scissors, and 7%–9% for spray bottles. We observed that this performance difference is caused mainly by their representation of human actions with incorrectly segmented atomic actions. From the experimental results, we see that our poselet representation of human actions, using a simple graphical model, is more effective at integrating human action information into object recognition.

**Figure 11:**
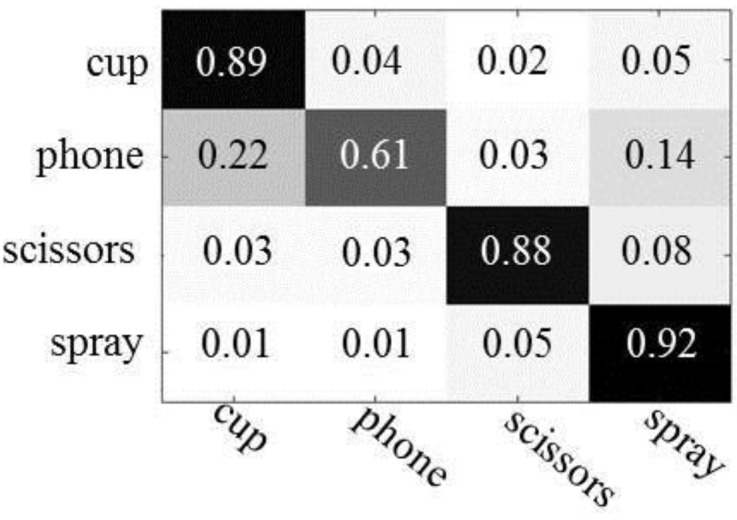
The results of applying the CNN to our experimental data.

**Figure 12:**
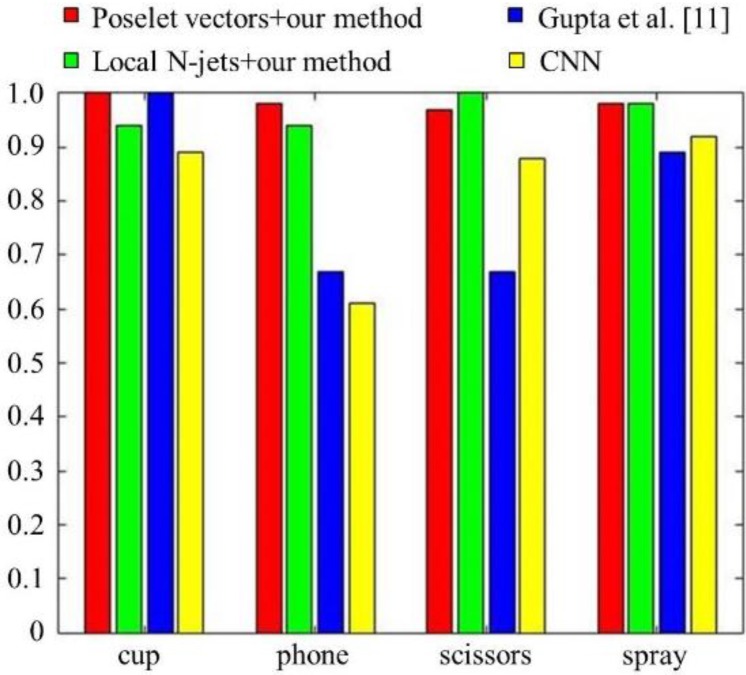
The performance comparison of our methods using poselet vectors and local N-jets with Gupta’s algorithm and CNN.

The results of applying the CNN to our experimental data are shown in Figure 11. To train the CNN, we used the same number of images for each category as was used in our method (1800). It can be seen that the recognition performance of our method outperforms the CNN. The performance improvements over CNN were 11% for cups, 34%–37% for telephones, 3%–10% for scissors, and 4%–6% for spray bottles. To allow for a clearer performance comparison, we also included Figure 12. We observed that 1800 labeled images for each category are not enough to adequately train the CNN and guarantee better performance than what was obtained by our method. Moreover, it is difficult to find the optimal CNN architecture for the given problem.

Cups and spray bottles look similar to each other, especially when they are held in a human hand, because of their cylindrical structure. Even some phones, such as cordless home phones, have appearances that are similar to cups and spray bottles in the feature space (due to their rectangular form). From the experimental results, we confirmed that our method greatly facilitates distinction between similar looking objects from different categories by efficiently exploiting the action information associated with the objects.

## 5. Conclusions

This work focused on the efficient use of object-action context to resolve the inherent difficulty of object recognition caused by large intra-category appearance variations and inter-category appearance similarities. To accomplish this, we proposed a method that integrates how humans interact with objects into object recognition. The probabilities of objects and actions have been computed effectively using random forest and multi-class Adaboost algorithms. Through experiments, we confirmed that a few key poses provide sufficient information for distinguishing human actions. When objects from different categories have similar appearances, the use of the human actions associated with each object can be effective in resolving ambiguities related to recognizing these objects. We also observed that when we have an insufficient amount of labelled objects, which inhibits recognition, carefully-designed statistical learning methods using handcrafted features are more adequate for obtaining an efficient solution, as compared to deep learning methods.

## Figures and Tables

**Table 1:** The CNN architecture used for the experiments.

	Operation	Input Size	Filter Size	Pool	Stride	Output Size
Layer1	Conv	50×50×3	5×5×3×32		1	50×50×32
Layer2	Max	50×50×32		3×3	2	25×25×32
Layer3	ReLU	25×25×32				25×25×32
Layer4	Conv	25×25×32	5×5×32×32		1	25×25×32
Layer5	ReLU	25×25×32				25×25×32
Layer6	Avg	25×25×32		3×3	2	12×12×32
Layer7	Conv	12×12×32	5×5×32×64		1	12×12×64
Layer8	ReLU	12×12×64				12×12×64
Layer9	Avg	12×12×64		3×3	2	6×6×64
Layer10	Conv	6×6×64	4×4×64×64		1	3×3×64
Layer11	fully-connected	3×3×64	3×3×64×64		1	1×1×64
Layer12	fully-connected	1×1×64	1×1×64×4		1	1×1×4
Layer13	Softmax	1×1×4				

## Abbreviations

The following abbreviations are used in this manuscript:

HMM	Hidden Markov Model
CNN	Convolutional Neural Network
SVM	Support Vector Machine
HOG	Histogram of Oriented Gradients
k-NN	k Nearest Neighbors
ReLU	Rectified Linear Unit
BoV	Bag of Visual words
STIP	Space Time Interest Point
